# Preclinical and clinical investigation of intratumoral chemotherapy pharmacokinetics in DIPG using gemcitabine

**DOI:** 10.1093/noajnl/vdaa021

**Published:** 2020-02-24

**Authors:** Adam L Green, Patrick Flannery, Todd C Hankinson, Brent O’Neill, Vladimir Amani, John DeSisto, Aaron Knox, Hannah Chatwin, Rakeb Lemma, Lindsey M Hoffman, Jean Mulcahy Levy, Jennifer Raybin, Molly Hemenway, Ahmed Gilani, Carl Koschmann, Nathan Dahl, Michael Handler, Angela Pierce, Sujatha Venkataraman, Nicholas Foreman, Rajeev Vibhakar, Michael F Wempe, Kathleen Dorris

**Affiliations:** 1 Morgan Adams Foundation Pediatric Brain Tumor Research Program, Aurora, Colorado, USA; 2 Center for Cancer and Blood Disorders, Children’s Hospital Colorado, Aurora, Colorado, USA; 3 Department of Pediatrics, University of Colorado School of Medicine, Aurora, Colorado, USA; 4 Department of Neurosurgery, University of Colorado School of Medicine, Aurora, Colorado, USA; 5 Department of Pathology, University of Colorado School of Medicine, Aurora, Colorado, USA; 6 University of Michigan, Ann Arbor, Michigan, USA; 7 University of Colorado School of Pharmacy and Pharmaceutical Sciences, Aurora, Colorado, USA

**Keywords:** chemotherapy, clinical trial, DIPG, PDX, pharmacokinetics

## Abstract

**Background:**

Hundreds of systemic chemotherapy trials in diffuse intrinsic pontine glioma (DIPG) have not improved survival, potentially due to lack of intratumoral penetration, which has not previously been assessed in humans.

**Methods:**

We used gemcitabine as a model agent to assess DIPG intratumoral pharmacokinetics (PK) using mass spectrometry.

**Results:**

In a phase 0 clinical trial of i.v. gemcitabine prior to biopsy in children newly diagnosed with DIPG by MRI, mean concentration in 4 biopsy cores in patient 1 (H3K27M diffuse midline glioma) was 7.65 µM. These compare favorably to levels for patient 2 (mean 3.85 µM, found to have an H3K27-wildtype low-grade glioma on histology), and from a similar study in adult glioblastoma (adjusted mean 3.48 µM). In orthotopic patient-derived xenograft (PDX) models of DIPG and H3K27M-wildtype pediatric glioblastoma, gemcitabine levels and clearance were similar in tumor, pons, and cortex and did not depend on H3K27 mutation status or tumor location. Normalized gemcitabine levels were similar in patient 1 and the DIPG PDX.

**Conclusions:**

These findings, while limited to one agent, provide preliminary evidence for the hypotheses that lack of intratumoral penetration is not why systemic chemotherapy has failed in DIPG, and orthotopic PDX models can adequately model intratumoral PK in human DIPG.

**Key Points**

- We tested whether chemotherapy can reach DIPG using clinical and mouse trials.
- Intravenous gemcitabine appears to reach DIPG tissue adequately for therapeutic response.
- Intratumoral PK from a DIPG PDX model closely matched the human PK.

**Importance of the Study**

DIPG is a universally fatal childhood brain tumor that has never been shown to respond to chemotherapy. We describe our clinical–translational research effort to begin to answer a crucial question in this field: Can systemic chemotherapy reach DIPG tissue? We report our chronologic work, from patient-derived cell culture to an immortalized orthotopic xenograft model, through results of our phase 0 clinical trial, and finally in orthotopic PDX models. We show that systemically administered gemcitabine achieves concentrations adequate for therapeutic effect and equal or greater to those observed in adult glioblastoma. We also demonstrate the fidelity of orthotopic PDX models in DIPG. While preliminary, given the single agent used and small patient numbers, this study provides the first human intratumoral pharmacokinetic evidence showing systemic chemotherapy could be part of the elusive goal of improving DIPG therapy.

Diffuse intrinsic pontine glioma (DIPG) is a highly aggressive and unresectable pediatric brain tumor that carries the worst prognosis of all childhood brain tumors, which as a category are the most common cause of death from pediatric cancer. Radiation therapy (RT) is effective in extending life but is not curative; the median overall survival is 11 months, and long-term survival is extremely rare.^1,2^ Hundreds of clinical trials of systemic chemotherapy, using both cytotoxic and targeted agents, single drug and combination approaches, and upfront and recurrent settings have failed to show any survival benefit over RT alone^1,3^ and, in fact, have caused significant toxicity to patients.^1^

One potential reason for the uniform failure of trials to date is a lack of penetration of systemically delivered chemotherapy into DIPG tissue. The blood-brain barrier (BBB), which serves to protect the central nervous system from harmful substances or organisms in the bloodstream, is well-established as a challenge in chemotherapy delivery.^4^ However, systemic chemotherapy has been shown to be effective in both adult and pediatric high-grade glioma (HGG) outside the brainstem,^5,6^ demonstrating that this potential issue with systemic chemotherapy is not common to all HGG. In addition, while one preclinical study showed poorer BBB penetration to pontine compared to cortical tumors based on MRI findings,^7^ systemic chemotherapy is also effective in pediatric low-grade brainstem gliomas,^1^ which shows that it is possible for systemic medications to reach brainstem tumors, even in low-grade gliomas (LGGs) in which the BBB is thought to be relatively intact compared to HGG.^8^ The question of systemic chemotherapy penetration to DIPG is pivotal, since if systemically administered chemotherapy does not effectively reach DIPG tissue, further systemic chemotherapy trials are likely futile, and local delivery methods should be prioritized.^9^ Accordingly, multiple DIPG reviews have called for an answer to this question.^10,11^

No published DIPG clinical trial has included intratumoral pharmacokinetic (PK) assessment, largely due to past concerns about the safety of biopsy in DIPG. Biopsy has now been proven safe in experienced hands,^12,13^ however, and is becoming more common. This has provided crucial tissue and cell lines for preclinical study. To attempt to answer the question of systemic chemotherapy penetration in DIPG, we chose gemcitabine as a model agent. Pediatric dosing and toxicity have been established in prior phase 1 trials,^14,15^ and the maximum tolerated dose in pediatric solid tumors has been established as 2100 mg/m^2^, given as a 30-min i.v. infusion.^14^ Most importantly, this cytidine analog, with a formal charge of 0 and a molecular weight of 263.2 g/mol, has been shown to cross the BBB and penetrate adult glioblastoma (GBM) tumors adequately for therapeutic effect,^16^ providing comparative data for our study.

We hypothesized that gemcitabine concentration in DIPG tissue would be lower than that observed in adult GBM and inadequate for antitumor activity. Here, we report the results of serial murine orthotopic xenograft and clinical trial studies. Our aims were (1) to investigate systemic chemotherapy penetration to DIPG tissue in laboratory models and patients, using gemcitabine as a model agent and (2) to measure the fidelity of mouse models to human subjects in measuring intratumoral PK in DIPG.

## Materials and Methods

### Cell Culture

Primary human pediatric DIPG/HGG cell lines (Supplementary Table S1) were grown as previously described (see Supplementary Methods for full description).^17^

### Orthotopic Xenografts

For cortical injections, the coordinates were 2.5 mm right and 2.0 mm anterior to bregma, then 3.5 mm below the skull surface. For pontine injections, the coordinates were 1.0 mm right and 0.8 mm posterior to lambda, then 5.0 mm below the skull surface. All mice were treated with gemcitabine 120 mg/kg i.p. Brains were then excised and divided into tumor (when applicable), normal pons, and normal cortex, then snap-frozen in dry ice (Supplementary Figure S1).

### Phase 0 Clinical Trial

Patients aged 3–18 years with newly diagnosed DIPG, based on clinical symptoms and brain MRI findings consistent with the diagnosis in the opinion of the local multidisciplinary neuro-oncology team, are eligible. The full study protocol is included in Supplementary Materials. Enrolled patients are administered gemcitabine 2100 mg/m^2^ i.v. over 30 min, with no more than 4 h allowed between the end of the infusion and obtaining of biopsy specimens to match the previous adult data and our mouse studies as closely as clinically feasible. Per institutional standard of care, 8 needle core biopsies were taken from 4 separate quadrants of the tumor’s circumference at two tissue depths for each quadrant. Four cores are available for study analysis only after clinical pathologic review is completed. A summary of the intratumoral drug concentration findings are discussed with the family once available (within 1 month of surgery) to help with the planning of subsequent therapy.

### Determination of Gemcitabine Concentration in Tissue Samples

An Applied Biosystems Sciex 4000 (Applied Biosystems) was equipped with a Shimadzu HPLC (Shimadzu Scientific Instruments, Inc.) and Leap auto-sampler (LEAP Technologies). Gemcitabine concentrations were determined using a liquid chromatography–mass spectrometry–mass spectrometry method employing a Thermo Scientific Hypersil Silica column (250 × 4.6 mm; 5 µm) run at 40^o^C with a flow rate of 0.4 mL/min.

### Statistical Analysis

Mean values for gemcitabine concentrations were compared via unpaired *t*-test. For dose–response curves, curve fitting and IC_50_ calculation were done via Graphpad Prism, and IC_90_ values were calculated at www.graphpad.com/quickcalcs/ECanything2/.

### Study Approval

The single-institution clinical trial was approved by the Colorado Multi-Institutional Review Board (COMIRB 15-1621, NCT02992015) at Children’s Hospital Colorado, and families of subjects completed informed consent before enrollment. Animal experiments were approved by the Institutional Animal Care and Use Committee at the University of Colorado Anschutz Medical Campus.

## Results

### Gemcitabine Is Effective in Patient-Derived Cell Culture Models of DIPG

**Figure 1.**
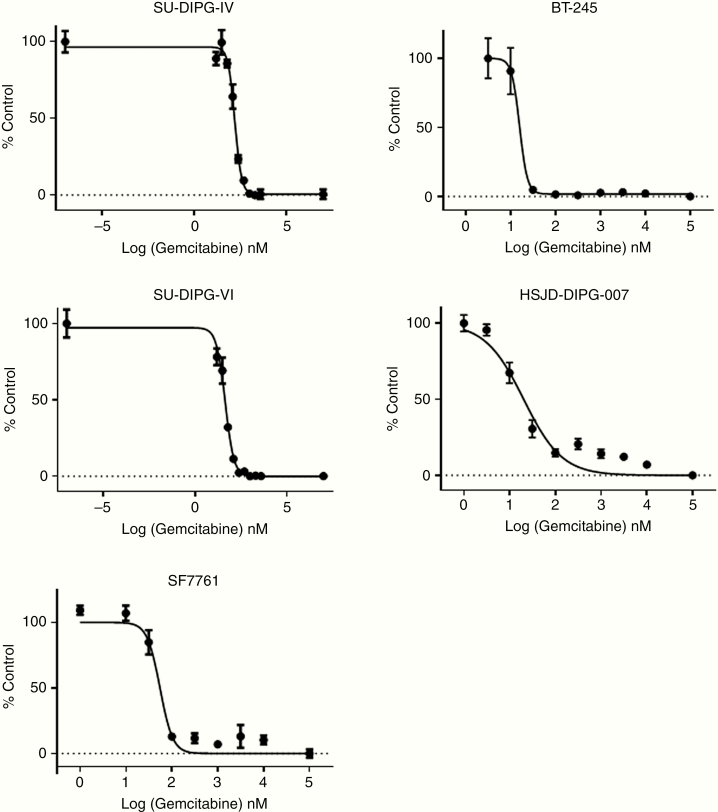
Dose–response curves of 5 primary patient-derived DIPG/DMG cells lines to gemcitabine (3 wells per condition). SU-DIPG-IV: IC_50_ 162 nM, IC_90_ 404 nM; SU-DIPG-VI: IC_50_ 44.3 nM, IC_90_ 147 nM; SF7761: IC_50_ 55.1 nM, IC_90_ 112 nM; BT-245: IC_50_ 15.8 nM, IC_90_ 24.6 nM; HSJD-DIPG-007: IC_50_ 20.4 nM, IC_90_ 171 nM. Error bars represent SEM.

To assess the antitumor efficacy of gemcitabine in DIPG, we first conducted dose–response experiments in 5 patient-derived cell culture models, including 3 autopsy-derived models (SU-DIPG-IV, SU-DIPG-VI, and HSJD-DIPG-007) and 2 biopsy-derived models (SF7761 and BT-245). Cells were treated for 3 or 5 days, and cell survival (viability as shown by metabolic activity) was then assayed by MTS. IC_50_ levels ranged from 15.8 to 162 nM (Figure 1) with low percentages of surviving cells at the highest drug concentrations. These results qualified gemcitabine for further study and established goal concentrations for potential treatment efficacy in vivo.

### Gemcitabine Concentration Is Decreased in Pontine Versus Cortical Tumors Using an Immortalized Adult GBM Model

**Figure 2.**
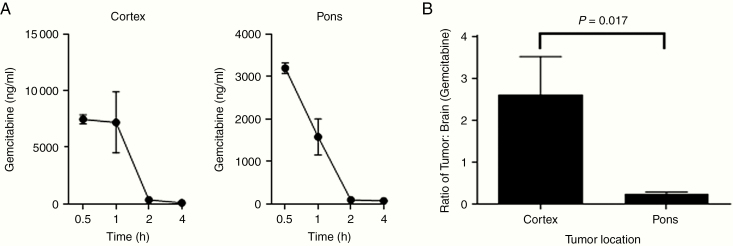
U87 model studies. (A) Gemcitabine concentration in normal mouse brain over time after a single 120 mg/kg i.p. dose (*n* = 2 per time point). (B) Comparison of gemcitabine tumor–normal brain concentration ratio 30 min after drug administration based on U87 xenograft tumor location (*n* = 5 per group). Error bars represent SEM.

Next, as an initial assessment of gemcitabine penetration based on tumor location, we used the immortalized adult GBM line, U87, to create orthotopic tumors in the cortex or pons of mice. We first determined, using nontumor-bearing mice, that the greatest gemcitabine levels occurred 30 min after i.p. injection and decreased thereafter (Figure 2A and B). Therefore, for tumor experiments, drug concentration was measured 30 min from injection. Five mice per group were injected in the pons or cortex with 100 000 U87 cells. Tumors were allowed to grow until mice became symptomatic, at which point they were treated with a single dose of gemcitabine 120 mg/kg i.p. and then sacrificed 30 min later. Tumors and normal brain were harvested, and gemcitabine concentration was measured. The ratio of gemcitabine concentration in tumor tissue compared to normal brain was significantly higher for cortical compared to pontine tumors (*P* = .017, Figure 2B). We concluded that in this immortalized xenograft model, chemotherapy was better able to penetrate cortical tumors than pontine tumors, supporting our study hypothesis and leading us to open a clinical trial.

### Initial Phase 0 Clinical Trial Data Show Adequate Gemcitabine Penetration to DIPG Tissue for Therapeutic Efficacy

**Figure 3.**
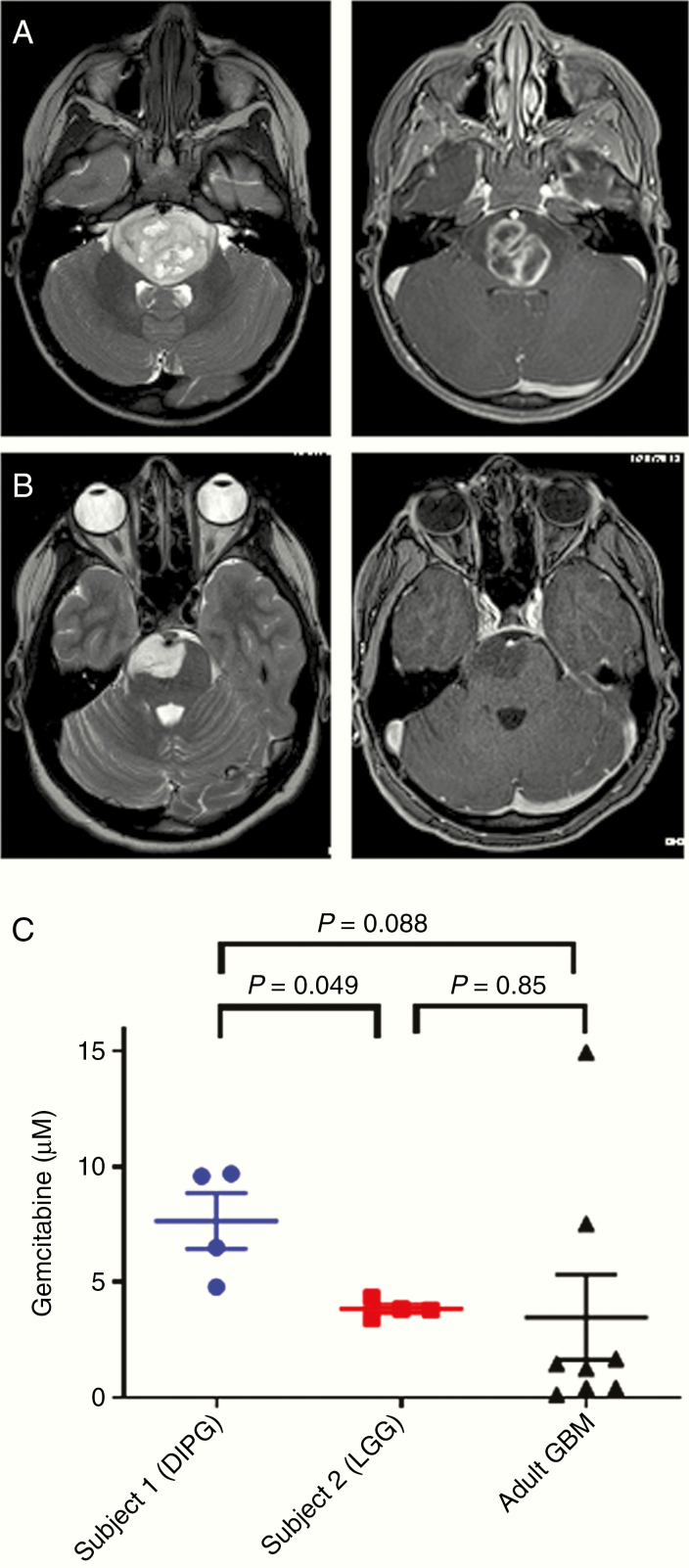
Clinical trial results. (A and B) T2 (left) and T1 post-contrast (right) axial magnetic resonance images of pontine tumors in patients 1 (A) and 2 (B). (C) Intratumoral gemcitabine concentrations in phase 0 clinical trial subjects compared to published values in adult glioblastoma patients. Error bars represent SEM.

Intratumoral PK of systemically administered gemcitabine in DIPG patients was evaluated through a phase 0 clinical trial. Patients received 1 dose of gemcitabine 2100 mg/m^2^ i.v. over 30 min and then underwent tumor biopsy as per institutional standard of care, with the actual removal of tumor tissue occurring approximately 2 h from the end of the infusion. Four tumor cores were sent for measurement of gemcitabine concentration by mass spectrometry. Two patients have been enrolled at this point. Patient 1 was 3 years old when she presented with 1 week of fatigue and drooling followed by 2 days of ataxia and aphasia. Initial MRI results are shown in Figure 3A and demonstrate a heterogeneous, expansile mass. Pathology showed a diffuse midline glioma (DMG), H3K27M-mutant, with a MIB-1 rate of 25–30%. She died approximately 16 months from diagnosis. Patient 2 was 15 years old when she presented with chronic headaches and then developed acute dizziness and tingling in her hands and feet. Initial MRI results are shown in Figure 3B; the tumor showed non-enhancing T2 hyperintense abnormal signal expanding the pons and partially surrounding the basilar artery, with a more focal area of T2 hyperintensity on the right, and was felt to be most consistent with DIPG. Pathology showed a low-grade infiltrating glioma (LGG), H3K27-wildtype, with a MIB-1 rate of 1–2%. She remains alive 2 years from diagnosis, and based on the overall clinical picture and pathology, she carries a diagnosis of brainstem LGG.

Mean gemcitabine concentration in the 4 biopsy cores for patient 1 (DIPG) was 7.65 µM (range 4.80–9.70), which was significantly greater than the mean concentration of 3.85 µM (range 3.42–4.33) for patient 2 (LGG) (*P* = .049, Figure 3C). It also compared favorably to the IC_50_ and IC_90_ levels measured for patient-derived DIPG cell lines (Figure 1). We then compared measured intratumoral gemcitabine levels from our patients to published concentrations in adult GBM^16^; in this adult trial, there was one level measured per patient. We adjusted these gemcitabine levels by a ratio based on the difference in doses between the trials (2100 mg/m^2^ in our trial compared to 500–1000 mg/m^2^ depending on the group in the adult trial).^16^ The mean adjusted gemcitabine level in the adult GBMs was 3.48 µM (range 0.12–14.96), which was not significantly different than the levels measured in either pediatric patient due to interpatient variability in the adult trial. These data indicate that, in our DIPG and LGG clinical trial subjects, adequate intratumoral drug penetration for potential antitumor effect was achieved.

### Gemcitabine Concentration Does Not Depend on Tumor/Brain Location or H3K27M Mutation Status in Orthotopic Patient-Derived Xenograft Models of Pediatric HGG

**Figure 4.**
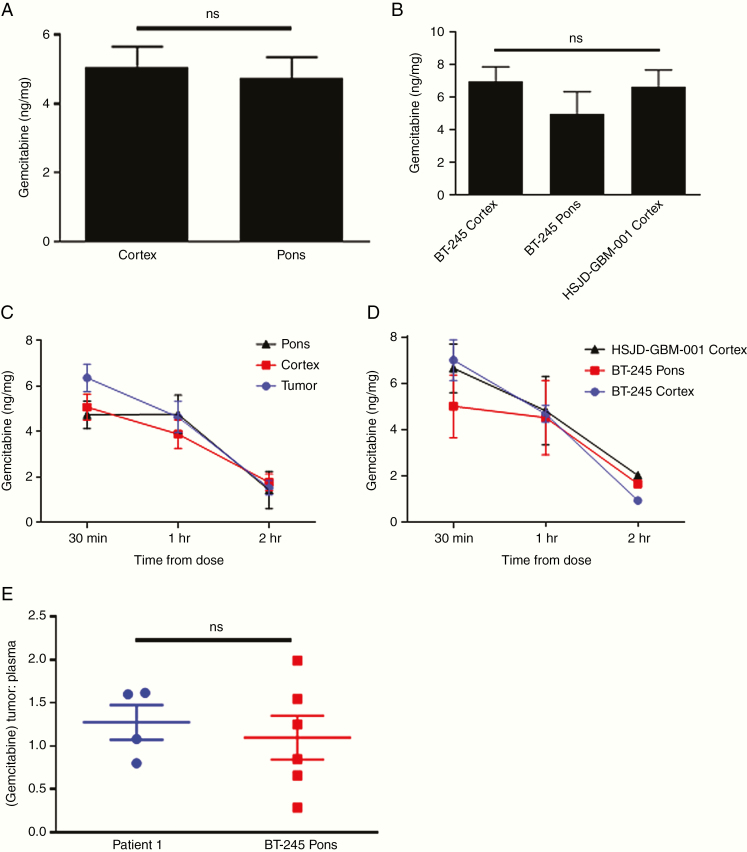
Orthotopic patient-derived xenograft (PDX) studies. (A) Comparison of gemcitabine concentration in normal mouse brain 30 min after drug administration based on location (*n* = 9 per group). (B) Comparison of gemcitabine concentration in murine orthotopic PDX tumors 30 min after drug administration based on location and H3K27 mutational status (*n* = 3 per group). (C) Comparison of gemcitabine concentration over time between PDX tumors and normal brain locations after a single i.p. dose (*n* = 6–9 per time point). (D) Comparison of gemcitabine concentration over time based on location and H3K27 mutational status after a single i.p. dose (*n* = 2–3 per time point). (E) Comparison of gemcitabine tumor–plasma concentration ratio between patient 1 and pontine BT-245 PDX tumors. Error bars represent SEM.

Given these initial clinical trial results, we returned to the laboratory to investigate whether gemcitabine penetration varies between brain and tumor locations or depending on the histone 3 mutational status of the tumor. To do this, we used 2 existing patient-derived cell lines to develop orthotopic patient-derived xenograft (PDX) models of pediatric HGG. Mice were injected with BT-245 (H3.3K27M-mutant pediatric DMG from Dr Keith Ligon, Dana-Farber Cancer Institute) cells in the pons (*n* = 8) or cortex (*n* = 8), or with HSJD-GBM-001 (H3K27-wildtype pediatric cortical GBM from Dr Angel Montero Carcaboso, Hospital Sant Joan de Deu) cells in the cortex (*n* = 8). Characteristics of the PDX models are shown in Supplementary Figure S1. As with the U87 model, mice were treated with one dose of gemcitabine i.p. at development of first symptoms and then sacrificed 30 min (*n* = 3 per group), 1 h (*n* = 3 per group), or 2 h (*n* = 2 per group) after treatment for measurement of gemcitabine concentration. Gemcitabine penetration to normal brain 30 min from administration in the cortex and pons was equivalent (Figure 4A). In tumor tissue harvested 30 min from administration, we also found no significant difference in levels based on tumor location (pons/cortex) or H3K27 mutation status (BT-245/HSJD-GBM-001) (Figure 4B).

Next, we tested whether tumor location or histone 3 mutation status could influence the rate of gemcitabine clearance by measuring drug levels over time. While gemcitabine levels decreased as expected with later time points, the rate of decrease was not significantly different in tumors compared to normal pons or cortex (Figure 4C), nor in tumors with different locations or H3K27 mutation status (Figure 4D). These data indicate that gemcitabine penetration and clearance are equivalent in normal brain and HGG tumors irrespective of location and mutation status.

### DIPG Clinical Trial and PDX Gemcitabine Concentration Are Similar When Normalized for Plasma Levels

Lastly, to test the fidelity of our PDX model to our phase 0 data from our DIPG subject, we compared gemcitabine levels in pontine H3K27M-mutant PDX tumors to those measured in patient 1. To control for differences in PK, intratumoral levels were normalized to plasma gemcitabine levels taken at the same time as tumor harvesting. The mean tumor to plasma ratios were 1.28:1 (range 0.80:1–1.62:1) for patient 1 and 1.10:1 (range 0.28:1–1.99:1) for the PDX tumors, and there was no significant difference between the levels overall (Figure 4E). Gemcitabine penetration in our DIPG subject thus appeared to be adequately modeled in our PDX tumors.

## Discussion

In this study, we investigated DIPG intratumoral PK using murine orthotopic xenografts, initially with an immortalized line and then with patient-derived cell lines, as well as a phase 0 clinical trial, using gemcitabine as a model compound. While our data from the U87 model that was available to us at the time suggested poorer drug penetration to pontine compared to cortical tumors, this finding was not borne out in subsequent clinical trial data or from the PDX models we developed subsequently. In these latter studies, gemcitabine reached DIPG tissue in adequate concentrations for therapeutic effect, based on in vitro dose–response curves. Recent phase 1/2 clinical trial data, however, demonstrate that gemcitabine does not impact survival in DIPG.^18^ This trial, in which gemcitabine was given weekly for 6 weeks, did use lower doses (140–200 mg/m^2^), which may account for the lack of effect. It is also important to acknowledge that intratumoral drug concentration alone is not enough to achieve antitumor efficacy, and that other factors, such as having adequate drug concentration over time and the necessity of gemcitabine to be converted to its active metabolite, may also explain this conflict. However, there may also be incompletely understood biological factors making human DIPG unresponsive to gemcitabine and other drugs that have been tried to this point.

Intratumoral PK was similar between our DIPG subject, brainstem LGG subject (whose pathology and clinical picture are less consistent with DIPG), and published adult GBM values. Similarly, in the PDX model, intratumoral PK was not significantly different based on tumor location or presence of the H3K27M mutation, which characterizes the WHO diagnosis of DMG. This result on tumor location is in conflict with our U87 data and with a prior study that used dynamic contrast-enhanced MRI in a genetically engineered mouse model of DIPG to demonstrate that BBB penetration was poorer in pontine compared to cortical tumors, although these authors also found no difference based on H3K27 mutational status.^7^ Our results also did not show more rapid clearance of gemcitabine from tumor compared to normal brain tissue. Finally, the pontine H3K27M-mutant model showed good fidelity in modeling intratumoral PK in our human DIPG subject. These first direct measurements of intratumoral PK in human DIPG, while preliminary and limited to a single agent, suggest that systemic chemotherapy has the potential for efficacy and can be accurately studied in PDX models.

Strengths of our study include the use of a clinical trial to investigate a hypothesis derived from a laboratory model, followed by the use of more advanced orthotopic PDX models to further investigate the clinical trial findings, all using the same mass spectrometry assay for PK measurement. The BT-245 DMG model and HSJD-GBM-001 H3K27-wildtype pediatric HGG orthotopic PDX models, which form tumors approximately 40 days from cell injection, should be useful for future preclinical studies in these diseases, especially given the fast timing of tumor development. Weaknesses include the absence of a pharmacodynamic marker for gemcitabine, small numbers of mice per time point especially at 2 h, and the early stage of the clinical trial data. With the development of therapeutic clinical trials open to newly diagnosed DIPG patients, accrual to a non-therapeutic trial like ours is challenging, although enrolled families have valued the data on tumor penetration for their discussions as to which routes of treatment to consider for subsequent therapy. Our trial remains open, with an accrual goal of 5 subjects. We plan to publish subsequent findings from these remaining patients that will include measures of gemcitabine’s active metabolite, gemcitabine triphosphate, as well as measures of unbound drug concentration, an important metric in brain tumor PK.^19^

We hypothesize, since our findings provide preliminary evidence using gemcitabine that systemic chemotherapy has the potential to penetrate DIPG tissue, that the unique biology of the disease,^20^ as opposed to a failure of chemotherapy to reach the tumor, may explain the lack of discovery of any effective agent to date despite the variety of drugs used in clinical trials. The most promising clinical agent found to address the epigenetic reprogramming in DIPG in preclinical models so far, panobinostat,^21^ unfortunately does not cross the BBB.^22^ Thus, we believe systemic chemotherapy trials should continue, focused on agents addressing H3K27M and its associated downstream effects. We anticipate that locally delivered therapy and RT will also ultimately be important in a multi-pronged approach to gain control of DIPG, given its devastating effects locally, in addition to its metastatic spread.^23^ Future early phase trials could incorporate intratumoral PK, both in orthotopic PDX models prior to opening, and in human subjects after safety/toxicity are established but before investigation of efficacy. This would allow objective assessment of drug concentration so a specific agent can be thoroughly vetted before proceeding with a larger trial. This approach would ensure a greater focus on drugs that have a chance at achieving better outcomes for patients with this otherwise terminal disease.

## Funding

This work was directly supported by grants from CureSearch, The Cure Starts Now, and the National Institute of Neurological Disorders and Stroke [1K08 NS102532-01], all to A.L.G., who also had early career support over this period from St. Baldrick’s Foundation/Luke’s Army and Hyundai Hope on Wheels.

## Supplementary Material

vdaa021_suppl_Supplementary_Table_S1

vdaa021_suppl_Supplementary_Figure_S1

vdaa021_suppl_Supplementary_Methods

vdaa021_suppl_Supplementary_Material

## Notes

**Authors’ Contributions.** A.L.G. conceived the study and led data analysis and manuscript writing. A.L.G., R.V., and K.D. planned the study and clinical trial. P.F. performed the mouse experiments and contributed to data analysis. M.W. performed PK experiments and analysis. T.C.H. and B.O. performed surgeries and procured clinical trial samples. L.M.H., J.M.L., C.K., A.P., S.J., M.H., and N.F. advised on study/experimental design and contributed to data analysis. V.A., J.D., A.K., H.C., and R.L. contributed to in vitro experiments and data analysis. A.G. and N.D. provided histological images. All authors contributed to manuscript revision.
