# Supplementary material for: Preclinical and clinical investigation of intratumoral chemotherapy pharmacokinetics in DIPG using gemcitabine
Source: Neurooncol Adv. 2020 Feb 24;2(1):vdaa021. doi: 10.1093/noajnl/vdaa021 (PMC7212907; doi:10.1093/noajnl/vdaa021)
Supplement: vdaa021_suppl_Supplementary_Table_S1 [file vdaa021_suppl_supplementary_table_s1.docx]

Table I: Cell Line Characteristics
