# Supplementary figures and images for: Preclinical and clinical investigation of intratumoral chemotherapy pharmacokinetics in DIPG using gemcitabine
Source: Neurooncol Adv. 2020 Feb 24;2(1):vdaa021. doi: 10.1093/noajnl/vdaa021 (PMC7212907; doi:10.1093/noajnl/vdaa021)

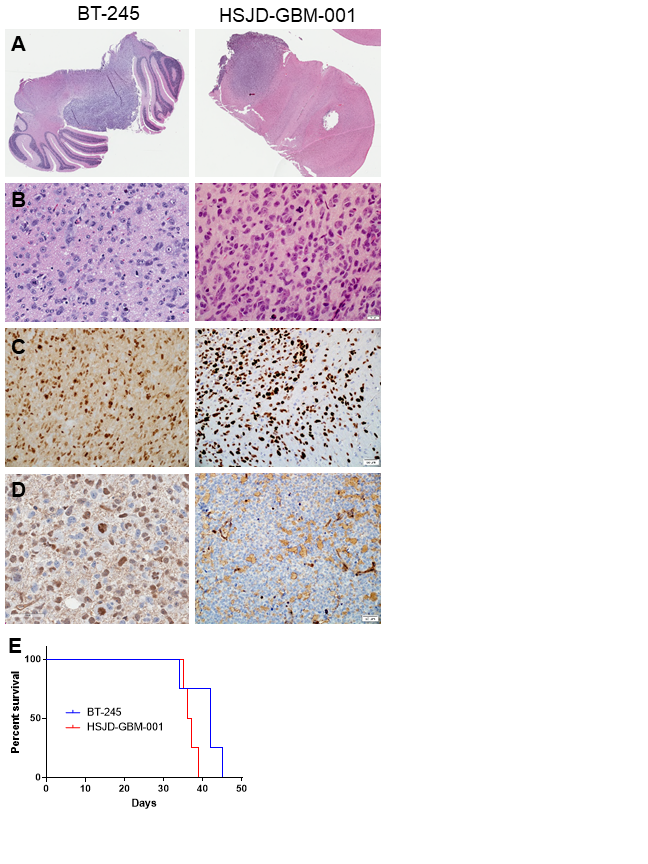

Supplement: vdaa021_suppl_Supplementary_Figure_S1 [file vdaa021_suppl_supplementary_figure_s1.png]
