# Supplementary material for: Preclinical and clinical investigation of intratumoral chemotherapy pharmacokinetics in DIPG using gemcitabine
Source: Neurooncol Adv. 2020 Feb 24;2(1):vdaa021. doi: 10.1093/noajnl/vdaa021 (PMC7212907; doi:10.1093/noajnl/vdaa021)
Supplement: vdaa021_suppl_Supplementary_Methods [file vdaa021_suppl_supplementary_methods.docx]

| 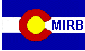 | COMIRB Protocol |  |
| --- | --- | --- |
| COLORADO MULTIPLE INSTITUTIONAL REVIEW BOARD  CAMPUS BOX F-490 TELEPHONE: 303-724-1055 Fax: 303-724-0990 | |  |

**Project Title: A Phase 0 Trial of Gemcitabine in Newly-Diagnosed Diffuse Midline Glioma**

**Protocol #: 15-1621**

**Version Date: October 30, 2018**

**Principal Investigator: Adam Green, MD** [adam.green@ucdenver.edu](mailto:adam.green@ucdenver.edu)

**Co-Investigators:** Kathleen Dorris, MD [kathleen.dorris@childrenscolorado.org](mailto:kathleen.dorris@childrenscolorado.org)

Rajeev Vibhakar, MD, PhD [rajeev.vibhakar@ucdenver.edu](mailto:rajeev.vibhakar@ucdenver.edu) Jean Mulcahy Levy, MD [jean.mulcahy-levy@childrenscolorado.org](mailto:jean.mulcahy-levy@childrenscolorado.org)

Nicholas Foreman, MD [nicholas.foreman@childrenscolorado.org](mailto:nicholas.foreman@childrenscolorado.org)

Lindsey Hoffman, DO [lindsey.hoffman@childrenscolorado.org](mailto:lindsey.hoffman@childrenscolorado.org)

Todd Hankinson, MD [todd.hankinson@childrenscolorado.org](mailto:todd.hankinson@childrenscolorado.org)

Michael Handler, MD [michael.handler@childrenscolorado.org](mailto:michael.handler@childrenscolorado.org)

Michael Wempe, PhD [michael.wempe@ucdenver.edu](mailto:michael.wempe@ucdenver.edu)

Margaret Macy, MD [margaret.macy@childrenscolorado.org](mailto:margaret.macy@childrenscolorado.org)

Molly Hemenway, PNP [molly.hemenway@childrenscolorado.org](mailto:molly.hemenway@childrenscolorado.org)

Corbett Wilkinson, MD [charles.wilkinson@childrenscolorado.org](mailto:charles.wilkinson@childrenscolorado.org)

Brent O’Neill, MD brent.o’neill@childrenscolorado.org

Allyson Alexander, MD [allyson.alexander@childrenscolorado.org](mailto:allyson.alexander@childrenscolorado.org)

**Clinical Research Coordinator:**

Elizabeth Chick, RN

Children’s Hospital Colorado

13123 East 16^th^ Avenue, B115

Aurora, CO 80045

Phone: (720) 777-3214

Fax: (720) 777-7289

Email: [elizabeth.chick@childrenscolorado.org](mailto:elizabeth.chick@childrenscolorado.org)

**Biology Laboratory:** Wempe Laboratory

Anschutz Medical Campus

Pharmacy and Pharmaceutical Sciences Building (V20)
 Second Floor, Room 2410A

12850 E. Montview Blvd

Aurora, CO 80045

Table of Contents

[1 OBJECTIVES 4](#_Toc528922159)

[1.1. Specific Aims 4](#_Toc528922161)

[1.2. Primary Objective 4](#_Toc528922162)

[2 BACKGROUND 4](#_Toc528922163)

[2.1. Rationale for Use of Gemcitabine (Including Preliminary Studies) 5](#_Toc528922164)

[2.2. Gemcitabine Metabolism 5](#_Toc528922165)

[2.3. Rationale for the Selected Approach and Trial Design 6](#_Toc528922166)

[3 RESEARCH METHODS 6](#_Toc528922167)

[3.1. Outcome Measures 7](#_Toc528922168)

[3.2. Pharmacokinetic Testing 7](#_Toc528922169)

[4 DESCRIPTION OF POPULATION TO BE ENROLLED 7](#_Toc528922170)

[4.1. Inclusion Criteria 8](#_Toc528922171)

[4.2. Exclusion Criteria 9](#_Toc528922172)

[5 Participant Registration 10](#_Toc528922173)

[5.1. Reserving a slot 10](#_Toc528922174)

[5.2. Registration Process 10](#_Toc528922175)

[6 Treatment Plan 11](#_Toc528922176)

[6.1. Gemcitabine Therapy 11](#_Toc528922177)

[6.2. Surgical Resection 11](#_Toc528922178)

[6.3. Treatment Post-Surgery 12](#_Toc528922179)

[6.4. Required Clinical, Laboratory and Disease Evaluations 12](#_Toc528922180)

[6.5. Supportive Care 13](#_Toc528922181)

[6.6. Duration of Therapy 13](#_Toc528922182)

[6.7. Duration of Follow-up 13](#_Toc528922183)

[6.8. Off Study Criteria 13](#_Toc528922184)

[7 SPECIMEN COLLECTION, HANDLING, LABELING AND SHIPPING INSTRUCTIONS 14](#_Toc528922188)

[7.1. Tumor Sample 14](#_Toc528922189)

[7.2. Peripheral Blood 14](#_Toc528922190)

[7.3. CSF 15](#_Toc528922191)

[7.4. Sample Labeling 15](#_Toc528922192)

[7.5. Sample Shipping: 15](#_Toc528922193)

[8 DRUG INFORMATION 15](#_Toc528922194)

[8.1. Mode of Action 16](#_Toc528922195)

[8.2. Dosage: 16](#_Toc528922196)

[8.3. Route of Delivery 16](#_Toc528922197)

[8.4. Adjustment of Dosing 16](#_Toc528922198)

[8.5. Availability 16](#_Toc528922199)

[8.6. Accountability 16](#_Toc528922200)

[8.7. Incompatibilities 17](#_Toc528922201)

[8.8. Toxicity 17](#_Toc528922202)

[8.9. Drug Preparation 17](#_Toc528922203)

[8.10. Storage and Stability 17](#_Toc528922204)

[9 EVALUATION CRITERIA 18](#_Toc528922205)

[9.1. Common Terminology Criteria for Adverse Events (CTCAE) 18](#_Toc528922206)

[10 ADVERSE EVENT REPORTING REQUIREMENTS 18](#_Toc528922207)

[10.1. Adverse Event (AE) Definition 18](#_Toc528922208)

[10.2. Non-Hematologic Adverse Events – Reporting Requirements 19](#_Toc528922209)

[10.3. Hematologic Adverse Events 0 Reporting Requirements 19](#_Toc528922210)

[10.4. Expectedness of Adverse Events 20](#_Toc528922211)

[10.5. Unanticipated Problems (UAPs) 20](#_Toc528922212)

[10.6. Serious Adverse Events (SAEs) 20](#_Toc528922213)

[11 REGULATORY REQUIREMENTS 21](#_Toc528922214)

[11.1. Protocol Review and Amendments 21](#_Toc528922215)

[11.2. Informed Consent 22](#_Toc528922216)

[11.3. Study Documentation 22](#_Toc528922217)

[12 DATA AND SAFETY MONITORING 22](#_Toc528922218)

[12.1. Data and Safety Monitoring Committee (DSMC) 22](#_Toc528922219)

[12.2. Data Collection 23](#_Toc528922220)

[13 DATA ANALYSIS PLAN 24](#_Toc528922224)

[14 KNOWLEDGE TO BE GAINED 24](#_Toc528922225)

[15 POTENTIAL SCIENTIFIC PROBLEMS 25](#_Toc528922226)

[15.1. Adequacy of Tissue Quantity for Analysis 25](#_Toc528922227)

[15.2. Appropriateness of Controls 25](#_Toc528922228)

[16 REFERENCES 26](#_Toc528922229)

# OBJECTIVES

## Specific Aims

The primary aim of this study is to determine the presence of gemcitabine in childhood diffuse midline glioma (DMG) tissue after systemic treatment with the drug.

The secondary aim is to quantify the intratumoral gemcitabine concentration after systemic treatment.

## Primary Objective

To measure the concentration of gemcitabine and its metabolites in patient biopsy-derived diffuse midline glioma tissue after pre-biopsy treatment and to compare these concentrations to historical control levels achieved in adult glioblastoma (GBM) tissue and to known *in vitro* inhibitory drug concentrations for diffuse midline gliomas, cell lines (previously classified as diffuse intrinsic pontine glioma (DIPG)).

# BACKGROUND

Most DIPG harbor the K27M mutation in either the HIST1H3B or H3F3A gene,(8) mutations which also occur in the majority of pediatric high-grade gliomas of the thalamus.(9) DMG is an aggressive childhood brain tumor that, despite many past clinical trials, has never been shown to respond to chemotherapy (1). These mutations now define a new WHO tumor category called diffuse midline glioma (DMG), which includes all midline tumors harboring a histone 3 K27M mutation.

Of note, before connection of these tumors through understanding of the mutation, midline gliomas outside the pons always underwent biopsy or resection. Radiation therapy (RT) is effective in extending life but is not curative; median overall survival is 11 months. It is still unclear why the hundreds of clinical trials involving chemotherapy for DMG have failed to demonstrate any activity against the tumor. Given that many agents tried in clinical trials cross the blood-brain barrier (BBB), it is possible that there are factors specific to DMG and its location that prevent adequate drug penetration. No previous clinical trial has included pharmacokinetic (PK) studies on tumor tissue to answer this question. Although there has historically been concern regarding the safety of biopsy of brainstem lesions, numerous publications have demonstrated that biopsy of DMG is safe in experienced hands (2-6). Furthermore, it is now considered standard of care at our institution to confirm histologic diagnosis due to the lack of reliability of diagnosis by imaging alone (7).

## Rationale for Use of Gemcitabine (Including Preliminary Studies)


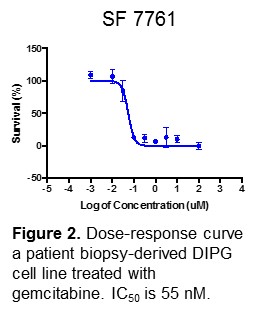

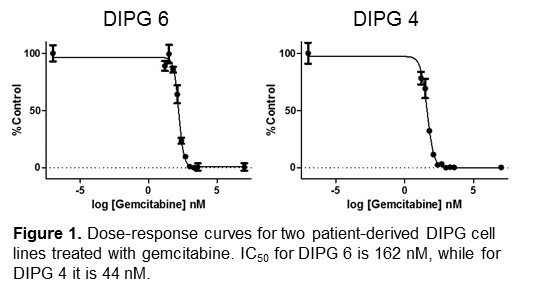
Gemcitabine was selected for this study because there is strong evidence of DMG cell line inhibition *in vitro* and good BBB penetration. Furthermore, pediatric dosing and toxicity has been established in prior studies of children with relapsed solid tumors and leukemia (10, 11). In the solid tumor study by Reid *et al,* the maximum tolerated dose for two consecutive week dosing was 2100 mg/m^2^, given as a 30-minute IV infusion. Gemcitabine has gone on to be studied in combination with multiple other agents in children, with some efficacy shown in phase 2 studies, largely in solid tumors (12-17). We found IC_50_ values of 44 and 162 nM for two pediatric patient autopsy-derived DMG cell lines treated with gemcitabine *in vitro* (Figure 1), and an IC_50_ of 55 nM for one pediatric patient biopsy-derived line (Figure 2). The drug has been shown to cross the BBB and penetrate adult glioblastoma (GBM) tumors (18). We subsequently treated athymic nude mice without tumors with a single dose of gemcitabine and then harvested their brains at set intervals. We then measured the concentration of gemcitabine in the pons and cortex of each mouse by mass spectrometry. The peak concentration of gemcitabine we observed in both parts of the brain occurred 30 minutes after administration, and the concentration in the pons was significantly less than that in the cortex (Figure 3A). I subsequently treated 5 cortical patient-derived xenograft (PDX) and 5 pontine PDX mice with one dose of gemcitabine upon first tumor symptoms and then harvested brains 30 minutes later. The ratio of tumor to normal brain gemcitabine concentration was significantly less for the pontine versus cortical tumors (Figure 3B).

## Gemcitabine Metabolism

Gemcitabine is a prodrug deoxynucleoside analogue that is phosphorylated intracellularly by deoxycytidine kinase to its active metabolite, gemcitabine triphosphate (dFdCTP), which competes for incorporation into DNA with the normal nucleotide deoxycytidine triphosphate. It is also deaminated to difluorodeoxyuridine (dFdU), which likely still has anticancer activity as a radiosensitizer (18). In an adult GBM study, dFdU was present at approximately 100 times the concentration of gemcitabine in tumor tissue, making it a very useful proxy measurement for gemcitabine concentration when using small tissue samples (18). In past studies, mass spectroscopy has been used to detect all three of these compounds in as little as 10 mg of tissue with a sensitivity of 0.2 ng/mg tissue (19).

This previous study involving adult GBM patients administered gemcitabine prior to biopsy/resection and then measured gemcitabine, dFdU, and dFdCTP levels in tumor. Those data provide a historical control analysis of gemcitabine in a similar tumor that will facilitate assessment of drug penetration into DMG tissue (18); the concentrations found were mostly above the limit of detection found in previous studies (19). This study will use analytic techniques that mirror those described previously in the adult study and will compare gemcitabine levels achieved to those achieved in the adult study, as a comparison of penetration between the tumor types, and to our *in vitro* IC_50_ values, as a measure of likelihood of efficacy.

## Rationale for the Selected Approach and Trial Design

The findings from this study will be crucial to defining the correct direction for DMG clinical trials so that the field can move toward prolonging high-quality life, and ultimately achieving a cure, while subjecting as few patients as possible to treatments that have no possibility of success. Thousands of DMG patients have been treated on clinical trials of systemic treatment over the last 40 years without any measurable benefit. Since limited amounts of biopsy tissue of DMG have been available, the understanding of the biology of the tumor is still evolving (20). Therefore, the prior failures of clinical trials may be more reflective of the incomplete knowledge of key driver mutations. However, the continued failure of more recent trials based on improved molecular understanding of DMG raises the question of whether systemically delivered chemotherapy adequately penetrates the DMG tumor. There is a shift in the pediatric neuro-oncology community to consider phase 0 studies in DMG before more large trials go forward. If gemcitabine can penetrate adequately into DMG, further study will be needed to evaluate its role in upfront treatment of DMG. If gemcitabine is able to penetrate the BBB but not DMG tumor tissue, especially if any future phase 0 trials of other agents show similar challenges with DMG tumor tissue penetration, these data would build a strong rationale for a refocusing of resources on alternative delivery methods, such as convection-enhanced delivery (21, 22), or intra-arterial therapy (23), which are currently at the very early stages of study. These data are essential to the effort to advance the care provided to these children with an otherwise uniformly terminal diagnosis.

# RESEARCH METHODS

This is a phase 0, multiple institution study of systemic gemcitabine in childhood DMG. We plan to enroll up to 5 evaluable patients on the study. If no gemcitabine is detected in the first 5 patients, then the sample size will be expanded to 10 patients. This will be done because we recognize that there is a chance that if the penetration rate is low, we might not detect gemcitabine in any patients in a sample of 5.

## Outcome Measures

Our outcome measures of interest are concentrations of gemcitabine, its metabolite difluorodeoxyuridine (dFdU), and gemcitabine nucleotides in peripheral blood and DMG tissue, both measured at the time of tumor biopsy/resection after a single IV dose of gemcitabine.

## Pharmacokinetic Testing

Levels of Gemcitabine and Metabolites in Tumor, Plasma, and CSF (only if accessed during surgery).

Description of Assay

Concentrations of gemcitabine, dFdU, and dFdCTP will be measured in tumor tissue, plasma, and CSF (when available) by liquid chromatography/tandem mass spectrometry (LC-MS/MS) in the UC-Denver Medical Chemistry core lab of Dr. Michael Wempe. An API 4000 instrument will be used, which has an approximate sensitivity of 0.1 ng/mg. Given the dimensions of the biopsy needle used, 10 mm length and 0.75 mm diameter, we expect that each core will have an approximate volume of 4.5 mm^3^, which translates to a likely mass of 4.5 mg (24). Given that approximately 10 mg of tumor tissue has been necessary for quantification of gemcitabine and its metabolites in past studies, multiple cores of the six available for quantification from neighboring regions of the tumor may be combined; whenever possible, however, more than one separate measurement of concentrations will be done for each patient to determine any variation in concentrations across the tumor. An internal control measurement for gemcitabine will be determined using a variant gemcitabine compound enriched with ^13^C.

# DESCRIPTION OF POPULATION TO BE ENROLLED

*Patient Eligibility Criteria*

All clinical and laboratory studies to determine eligibility must be performed within seven (7) days prior to enrollment unless otherwise indicated. Imaging studies must be obtained within 14 days of enrollment and 21 days of start of protocol therapy. The patient may begin protocol therapy once all pre-study requirements are met.

Important note: The eligibility criteria listed below are interpreted literally and cannot be waived. All clinical documentation required for determining eligibility of a patient enrolled on this trial must be available in the patient's medical/research record which will serve as the source document for verification at the time of audit.

## Inclusion Criteria

1. Age greater than or equal to 3 years and less than 18 years at the time of enrollment.

2. Patients must meet both of the first two conditions, OR the third:

- Clinical findings consistent with a presumed new diagnosis of diffuse midline glioma (DMG) in the opinion of the treating neuro-oncologist, AND
- Brain MRI findings consistent with a new diagnosis of DMG based on multidisciplinary consensus after review of imaging
- OR, recurrent DMG requiring tumor resection or biopsy

3. Organ Function Requirements

Adequate bone marrow function defined as:

- Platelet count ≥100,000/µl (no platelet transfusion for more than 3 days)
- Hemoglobin >8 g/dl and absolute neutrophil count (ANC) ≥1,000/µl

Adequate coagulation defined as:

- Prothrombin time (PT) and activated partial thromboplastin time (aPTT) ≤ upper limit of normal (ULN) for age

Adequate renal function defined as:

- Creatinine clearance or radioisotope GFR > 70 ml/min/1.73 m^2^ or
- A serum creatinine based on age/gender as follows:

| **Age** | **Maximum Serum Creatinine (mg/dL)** | |
| --- | --- | --- |
|  | **Male** | **Female** |
| 3 to < 6 years | 0.8 | 0.8 |
| 6 to < 10 years | 1 | 1 |
| 10 to < 13 years | 1.2 | 1.2 |
| 13 to < 16 years | 1.5 | 1.4 |
| 16 years to < 18 years | 1.7 | 1.4 |

The threshold creatinine values in this Table were derived from the Schwartz formula for estimating GFR (Schwartz et al. J. Peds, 106:522, 1985) utilizing child length and stature data published by the CDC.

Adequate liver function defined as:

- Total bilirubin <3x ULN for age and SGOT (AST) and SGPT (ALT) <2.5x ULN for age

4. Patients must meet one of the following performance scores:

- ECOG performance status scores of 0, 1, or 2;
- Karnofsky score of ≥ 60 for patients > 16 years of age; or
- Lansky score of ≥ 60 for patients ≤ 16 years of age

5. DMG biopsy/resection is planned for the clinical care of the patient independent of study participation by the treating pediatric neurosurgeon and neuro-oncologist.

6. Informed consent and assent obtained as appropriate.

## Exclusion Criteria

1. Pregnant or breastfeeding patients are not eligible due to teratogenic effects seen in animal/human studies.

2. Patients who have received any tumor-directed therapy prior to biopsy are not eligible. **Concurrent treatment with corticosteroids is allowed.**

3. Any tumor-specific or clinical features that make surgical intervention unsafe in the opinion of the treating neurosurgeon

4. Patients with personal or family history of bleeding disorders are not eligible.

5. Patients with uncontrolled intercurrent illness including, but not limited to ongoing or active infection, symptomatic congestive heart failure, unstable angina pectoris, cardiac arrhythmia, or psychiatric illness/social situations that would limit compliance with study requirements are not eligible.

# Participant Registration

## Reserving a slot

Prior to enrolling any participant on study, CHCO must be contacted to reserve a slot. A slot may be reserved by emailing the study coordinator at CHCO who will confirm slot availability via email. There are a total of 5 slots available and this number may potentially be increased to 10. Therefore, it is imperative that CHCO be contacted before the patient signs consent to see if there are any available slots and avoid over enrollment. Once confirmation of a slot reservation has been received, the participant may sign consent and the registration process may begin.

Enrollment: The participant will be considered enrolled on study once the consent is signed and the patient has been registered.

## Registration Process

Registration of all participants must occur before any study-related procedures. Staff will be available to register participants Monday thru Friday, from 8:00 AM to 5:00 PM Mountain Standard Time. In emergency situations, where a patient must be registered during holidays or off-hours, you may page or call the research coordinator using the information listed on the first page. You may not register a participant if a slot has not been reserved.

**Please follow these steps to register a participant**

1. Obtain written informed consent/assent prior any study related procedures.
2. Complete the eligibility checklist. The local investigator must sign the checklist confirming that eligibility has been reviewed.
3. Send the signed eligibility checklist along with supporting source documents, including the consent/assent, to the research coordinator at CHCO. You may send all the documents via fax or email. Please fax to 720-777-7289 or email the CHCO coordinator listed on the first page of the protocol.

1. An investigator at CHCO will review and confirm eligibility. The eligibility will be signed by the investigator and returned to the site. An email of registration confirmation will be sent to the overall study PI, local investigator and local study coordinator.
2. The participant will be assigned a participant number that includes the site number followed by the sequential order of participants enrolled locally; site #_sequential number (01_01 then 01_02 for site number 01)

# Treatment Plan

## Gemcitabine Therapy

The entire therapy on this study is one dose of gemcitabine. No intrapatient dose modifications are necessary. Gemcitabine will be given at 2100 mg/m^2^ IV over 30 minutes (+/- 10 minutes) within 4 hours of planned surgical procedure. The four hour time point begins at the end of the infusion. If delays in operation occur after gemcitabine is given such that the procedure occurs 4-12 hours after the end of the gemcitabine infusion is given, the patient can remain on study and tissue will be collected. If the surgery is delayed more than 12 hours after the end of the gemcitabine infusion is given, the patient will come off study and the tissue will be collected for clinical diagnostic purposes only.

## Surgical Resection

Patients enrolled will have a biopsy or resection as part of their standard-of-care therapy. It is important to note that participants will not undergo surgical resection for the sole purpose of participation on this study. The exact surgical location and number of samples will be determined by the treating neurosurgeon with the goal of minimizing procedural risk. The standard-of-care surgical procedure at CHCO involves 8 needle cores, taken from four separate quadrants of the tumor’s circumference, and two separate depths. Tumor tissue from this standard-of-care surgical procedure will be collected and sent to pathology for histologic diagnosis/further clinical testing (2 cores) and Wempe Laboratory for PK analysis (6 cores). Of note, tumor samples for PK analysis should be snap frozen within 30 minutes of resection and then maintained in freezer at -80° C until processing. Peripheral blood will be drawn at the end of gemcitabine infusion and at the time of the biopsy collection and placed on ice. Please see section 7 of the protocol for details of collections. Patient biopsy/resection will be planned as clinically indicated, and gemcitabine treatment will be planned around surgery timing.

## Treatment Post-Surgery

Enrolled patients’ post-surgical treatment will be per standard of care as determined by treating neuro-oncologist. That treatment may proceed even when patient remains in the follow-up period for this study.

## Required Clinical, Laboratory and Disease Evaluations

All clinical and laboratory studies to determine eligibility must be performed within seven (7) days prior to enrollment unless otherwise indicated. Laboratory values used to assess eligibility (see Section 4.1) must be no older than seven (7) days at the start of chemotherapy. Imaging studies must be obtained within 14 days prior to enrollment and 21 days from start of protocol therapy. The start of protocol therapy is defined as the initiation of gemcitabine.

| **Observation** | **Pre-Study** | **Surgery Period^1^** | **Follow-up Period^7^** |
| --- | --- | --- | --- |
| History | X | X | X^8^ |
| Physical exam with vital signs | X | X | X^8^ |
| Performance status | X |  | X^8^ |
| CBC with differential | X | X^4^ |  |
| PT, PTT, INR | X |  |  |
| Comprehensive metabolic panel | X |  |  |
| Pregnancy test^2^ | X |  |  |
| MRI Brain with and without contrast^3^ | X |  |  |
| MRI Spine with contrast^3^ | X |  |  |
| Gemcitabine administration |  | X |  |
| Tumor Specimen |  | X |  |
| CSF Specimen |  | X^5^ |  |
| Blood specimen |  | X^6^ |  |

^1^Begins at time of gemcitabine infusion and continues through Post-Operative Day (POD) 14. AEs should be assessed and documented with every patient interaction that occurs (using the CTCAE v4.0)

^2^For females of childbearing potential

^3^MRI should be within 21 days of gemcitabine/surgery; spine MRI required if clinically indicated

^4^CBC should be obtained as per standard of care. Transfusion requirements are included in Section 6.5.

^5^Sample optional and should only be obtained if the surgeon will be accessing the ventricular space at time of surgery (e.g., for ventriculoperitoneal shunt placement).

^6^Two blood specimen should be drawn. The first sample at the end of gemcitabine infusion and the second sample in the OR and stored/processed per Section 7.2.

^7^Defined as POD14-30

^8^Visits should be every week

## Supportive Care

Given the proximity of gemcitabine treatment to surgery, we do not expect any significant myelosuppressive effect of the drug at the time of surgery (4 hours after the end of infusion). Patients will be closely monitored for signs of bleeding post-operatively through anticipatory guidance for family care at home to monitor for signs such as worsening headache, and through physical exams according to the schedule in Section 6.4. We will minimize the risk of post-operative hemorrhage by monitoring CBCs as per section 6.4 transfusing to maintain platelet counts greater than 50,000/µl for two weeks after surgery.

For patients who decide to proceed with radiation therapy, it is important to note that radiation therapy should not begin any earlier than 10 days from the gemcitabine infusion.

## Duration of Therapy

The only therapy administered will be the pre-surgical dose of gemcitabine.

## Duration of Follow-up

Adverse events will be collected for 30 days after the completion of the pre-surgical gemcitabine. No clinical follow up will be obtained after the 30-day toxicity window.

## Off Study Criteria

- 30 days after the gemcitabine dose
- Death
- Loss to follow-up
- Withdrawal of consent for any further required observations or data submission
- Surgery is delayed more than 12 hours after the gemcitabine is given

# SPECIMEN COLLECTION, HANDLING, LABELING AND SHIPPING INSTRUCTIONS

## Tumor Sample

Submission of tumor tissue sample is an essential part of this protocol and is required. Tumor samples should be collected at the time of surgery and snap frozen within 30 minutes of resection. Submission of tumor tissue sample is a required part of the study. Tumor samples should be collected at the time of surgery and snap frozen within 30 minutes of resection. Two cores should be processed through local pathology as per clinical routine for fixation. The remaining cores (ideally 6) should be snap frozen for use under this protocol. The frozen samples should be maintained in a -80° C freezer until shipment on dry ice to the Wempe Laboratory. The Wempe lab should be notified prior to any shipments to confirm receipt.

Sample collection form will be included with all samples being sent to the Wempe laboratory.

## Peripheral Blood

Submission of peripheral blood sample for plasma extraction is a required part of the study. Two samples of peripheral blood will be drawn. One 2.5 mL sample at the end of gemcitabine infusion and one 2.5mL sample at the time of the biopsy. Collect whole blood into commercially available anticoagulant-treated tubes; EDTA-treated (lavender tops) and place on ice. Cells are removed from plasma by centrifugation for 15 minutes at 2,000 x g using a refrigerated centrifuge. The resulting supernatant is designated plasma. Following centrifugation, it is important to immediately transfer the liquid component (plasma) into a clean polypropylene tube using a Pasteur pipette. The samples should be maintained at 2–8°C while handling, including centrifugation. The plasma should be apportioned into 0.5 ml aliquots, stored, and transported at –20°C or lower. If samples are to be maintained on site for shipment at a later date, place samples in a -80°C freezer until shipment on dry ice to the Wempe Laboratory.

## CSF

Submission of CSF is optional, and sample should only be obtained if accessible during the context of a clinically indicated procedure. A minimum of 100 micro liters of CSF should be collected. CSF will be aliquoted into cryovials after collection and placed in a -80°C freezer until shipment.

## Sample Labeling

Each specimen should be labeled with the patient ID number along with the date and time of the surgical resection/sample collection.

Patient ID: (patient #)

Date of Surgery: (mm/dd/yyyy)

Time of collection:

## Sample Shipping:

All samples will be sent to the Wempe Laboratory Monday thru Thursday. Please contact the laboratory before sending samples. Samples cannot be sent on a Friday or the day before a holiday. If they are collected on a Friday or over the weekend, please maintain samples until Monday for shipment.

**Wempe Laboratory**

Anschutz Medical Campus

Pharmacy and Pharmaceutical Sciences Building (V20)
Second Floor, Room 2410A

12850 E. Montview Blvd

Aurora, CO 80045

Telephone: (303) 724-8982

Email: [Michael.Wempe@ucdenver.edu](mailto:Michael.Wempe@ucdenver.edu)

# DRUG INFORMATION

**Names, chemical nature and classification:** Gemcitabine has the chemical name: 2'-deoxy-2', 2'-difluorocytidine monohydrochloride. Molecular formula C9H12ClF2N3O4; MW=299.66 daltons. Also known as LY188011. It has broad-spectrum antitumor activity against murine leukemias, murine solid tumors, and human tumor xenografts.

## Mode of Action

Gemcitabine has unique properties as an antimetabolite. The drug is cell-cycle specific in blocking cells at the G1/S interface and is retained in human tumor cells for long periods.

Physical description: It is supplied as a lyophilized product containing the equivalent of 200 mg or 1000 mg gemcitabine in vials.

## Dosage:

Gemcitabine will be given at 2100 mg/m^2^ IV over 30 minutes within 4 hours of planned surgical procedure. The four hour time point begins at the end of the infusion. If delays in operation occur after gemcitabine is given such that the procedure occurs 4-12 hours after the end of the gemcitabine infusion is given, the patient can remain on study and tissue will be collected. If the surgery is delayed more than 12 hours after the end of the gemcitabine infusion is given, the patient will come off study and the tissue will be collected for clinical diagnostic purposes only.

## Route of Delivery

Intravenous over 30 minutes within 4 hours of planned surgical procedure.

## Adjustment of Dosing

The entire therapy on this study is one dose of gemcitabine. No intrapatient dose modifications are necessary.

## Availability

Supplied as a lyophilized powder containing either 200 mg or 1000 mg of active drug gemcitibine as the hydrochloride salt, mannitol, and sodium acetate.

## Accountability

Dosing and time dosed will be recorded in the patient’s medical record.

## Incompatibilities

No incompatibilities with other drugs have been described. The drug is stable in normal saline as stated above.

## Toxicity

**Gastrointestinal:** nausea, vomiting, anorexia, diarrhea

**Blood:** Neutropenia, anemia, thrombocytopenia.

**Dermatologic:** transient mild erythematous, pruritic rash, desquamation.

**CNS:** somnolence, agitation, insomnia, dizziness, paresthesia, confusion, convulsion, coma.

**Renal:** renal dysfunction (increased BUN and creatinine values, proteinuria, or hematuria).

**Other:** Alopecia, mild chills and fever, flu-like symptoms, fatigue, weakness with or without myalgia, headache, shortness of breath, rare: hypotension.

## Drug Preparation

Reconstitution & Route of administration: To reconstitute, add 5 mL of 0.9% Sodium Chloride Injection to the 200 mg vial or 25 mL of 0.9% Sodium Chloride to the 1 gram vial. Shake to dissolve. These dilutions each yield a gemcitabine concentration of 38 mg/mL which includes accounting for the displacement volume of the lyophilized powder (0.26 mL for the 200 mg vial or 1.3 mL for the 1 g vial). The total volume upon reconstitution will be 5.26 mL or 26.3 mL, respectively. Complete withdrawal of the vial contents will provide 200 mg or 1 gram of gemcitabine, respectively. The reconstituted solution is stable for 24 hours at room temperature.

The reconstituted gemcitabine solution can be further diluted with 0.9% NaCl to a final concentration of 0.1 – 38 mg/mL. This dilution is stable for 24 hours at room temperature.Gemcitabine is not a vesicant, but the pH of the reconstituted solution is approximately 3. Without further dilution, this low pH may cause local irritation at the site of injection.

## Storage and Stability

The lyophilized compound should be stored at controlled room temperature, 15°-30° C (59°-86° F). The reconstituted vials contain no antibacterial preservative, use within 24 hours. No degradation of the drug product in the dry state (vials) has been observed after six months at 40 degrees C with 75% relative humidity or after three years at room temperature.

# EVALUATION CRITERIA

## Common Terminology Criteria for Adverse Events (CTCAE)

The descriptions and grading scales found in the revised NCI Common Terminology Criteria for Adverse Events (CTCAE) version 4.0 will be utilized for AE reporting. All appropriate treatment areas should have access to a copy of the CTCAE version 4.0. A copy of the CTCAE version 4.0 can be downloaded from the CTEP website (<http://ctep.cancer.gov>).

# ADVERSE EVENT REPORTING REQUIREMENTS

All adverse events (grade 1 and higher) that occur while the patient is on study will need to be recorded in the AE log specific to each patient. All AEs will be reported to the DSMC for safety every 6 months. Please note that some AEs require expedited reporting. The expectations for expedited reporting are listed in more detail in the following sections.

There are specific reporting requirements for non-hematologic and hematologic AEs. Please see below for specific requirements. An AE log will be maintained in order to capture all AEs and SAEs.

## Adverse Event (AE) Definition

An AE is any unfavorable and unintended sign, symptom, or disease temporally associated with the use of an investigational medicinal product (IMP) or other protocol-imposed intervention, regardless of attribution. Safety assessments will consist of monitoring and reporting AEs and serious adverse events (SAEs), which includes all events of death and any study-specific issue of concern as outlined below. All AEs (including grade 1) will be recorded in the AE log for each patient.

**AEs may include the following:**

- AEs not previously observed in the subject that emerge during the protocol-specified AE reporting period, including signs or symptoms associated with DMG that were not present prior to the AE reporting period.
- Complications that occur as a result of protocol-mandated interventions
- Preexisting medical conditions (other than the condition being studied) judged by the investigator to have worsened in severity or frequency or changed in character during the protocol-specified AE reporting period.

All adverse events will be evaluated according to the NCI Common Terminology Criteria for Adverse Events (CTCAE) v4.0.

## Non-Hematologic Adverse Events – Reporting Requirements

Non-hematologic adverse events will be monitored starting at the time the participant signs consent and ending 30 days after the participant’s last dose of gemcitabine. All toxicities, including Grade 1 or higher will be recorded in the AE log and sent to the CHCO coordinator. All AEs will be reported to the DSMC every 6 months for safety review.

Please see below for AEs that need to be reported within a determined time frame. All AEs (including grade 1) must still be recorded for reporting to our DSMC.

| **Attribution** | **Requirements for reporting Non-Hematologic AEs** | | | | |
| --- | --- | --- | --- | --- | --- |
|  | **Grade1, 2 & 3 AE**  **Expected** | **Grade 1, 2 & 3 AE**  **Unexpected** | **Grade 4**  **AE**  **Expected** | **Grade 4**  **AE Unexpected** | **Grade 5**  **AE**  **Expected or Unexpected** |
| **Unrelated**  **Unlikely** | Not required | Not required | Report within 3 days* | Report within 3 days* | Report within 24 hours* |
| **Possible**  **Probably**  **Definite** | Not required | Report within 3 days* | Report within 3 days* | Report within 3 days* | Report within 24 hours* |

*Please report these AEs to the PI within the allotted time frame so sufficient time is given for IRB and DSMC report completion.

## Hematologic Adverse Events 0 Reporting Requirements

Hematologic adverse events will also be monitored starting at the time the participant signs consent and ending 30 days after the participant’s last dose of gemcitabine. All toxicities, including Grade 1 or higher will be recorded in the AE log and sent to the CHCO coordinator. All AEs will be reported to the DSMC every 6 months for safety review.

Please see below for AEs that need to be reported within a determined time frame. All AEs (including grade 1) must still be recorded for reporting to our DSMC.

| **Attribution** | **Requirements for reporting Hematologic AEs** | | | | |
| --- | --- | --- | --- | --- | --- |
|  | **Grade 1, 2 & 3**  **AE**  **Expected** | **Grade 1, 2 & 3**  **AE**  **Unexpected** | **Grade 4 AE**  **Expected** | **Grade 4 AE Unexpected** | **Grade 5 AE**  **Expected or Unexpected** |
| **Unrelated**  **Unlikely** | Not required | Not required | Not required | Report within 3 days* | Report within 24 hours* |
| **Possible**  **Probably**  **Definite** | Not required | Report within 3 days* | Not required | Report within 3 days* | Report within 24 hours* |

*Please report these AEs to the PI within the allotted time frame so sufficient time is given for IRB and DSMC report completion.

## Expectedness of Adverse Events

An adverse event will be recorded as “expected” or “unexpected”. Depending on the grade of each AE and whether is it expected or unexpected, it will need to be reported to the DSMC. Please see the tables above for reporting requirements.

## Unanticipated Problems (UAPs)

Unanticipated Problems (UAPs) include adverse events which in the opinion of the principal investigator are

- Both unexpected and possibly, probably or definitely related to the intervention/ drug or device
- Any unforeseen development that potentially increases the likelihood of harm to participants or others in the future
- Information that indicates a change to the risks or potential benefits of the research or an actual unforeseen harmful or unfavorable occurrence to participants or others that relates to the research protocol (injuries, psychological events, drug errors).

All UAPs must be reported to CHCO for reporting to the IRB of record and the DSMC within 5 business days of receiving notification of the occurrence. Please follow the guidance of the tables above to determine when an AE needs to be reported. Any AE listed in the tables above as having to be reported will qualify as a UAP.

## Serious Adverse Events (SAEs)

An Adverse Event is considered serious (SAE) if ANY of the following criteria are met:

1. It results in Death (i.e., the AE actually causes or leads to death)
2. It is life-threatening (i.e., the AE, in the view of the investigator, places the subject at immediate risk of death. It does not include an AE that, had it occurred in a more severe form, might have caused death.)
3. It requires or prolongs inpatient hospitalization.
4. It results in persistent or significant disability/incapacity (i.e., the AE results in substantial disruption of the subject’s ability to conduct normal life functions).
5. It results in a congenital anomaly/birth defect in a neonate/infant born to a mother exposed to gemcitabine
6. It is considered a significant medical event by the investigator based on medical judgment (e.g., may jeopardize the subject or may require medical/surgical intervention to prevent one of the outcomes listed above).

Important medical events that may not result in death, be life-threatening, or require hospitalization may be considered serious when, based upon appropriate medical judgment, they may jeopardize the patient or subject and may require medical or surgical intervention to prevent one of the outcomes listed in this definition.

Food and Drug Administration (FDA) REPORTING REQUIREMENTS FOR SERIOUS ADVERSE EVENTS (21 CFR Part 312)

NOTE: Investigators MUST immediately report to the sponsor (University of Colorado/CHCO) ANY Serious Adverse Events that occur within 30 days of last gemcitabine dose, whether or not they are considered related to the investigational agent. If a patient is still being followed and an SAE occurs more than 30 days after last administration of gemcitabine and has an attribution to gemcitabine of possible, probable or definite, the SAE must also be reported to the Sponsor/Principal Investigator within 24 hours of Awareness Date (date of first learning of event) (21 CFR 312.64).

All SAEs must be reported by the PI or designated study member, to the IRB of record and the DSMC within 5 business days of receiving notification of the occurrence.

# REGULATORY REQUIREMENTS

## Protocol Review and Amendments

This protocol, the proposed informed consent and assent forms along with any documents being given to participants (e.g. drug diaries) must be submitted, reviewed and approved by a properly constituted IRB.

Any changes made to the protocol will be submitted as amendments and must be approved by the COMIRB prior to implementation.

## Informed Consent

All participants must be provided a consent and assent form (if applicable) describing the study. All participants must be provided with sufficient information to make an informed decision about their participation in this study. The formal consent of a participant, using the IRB approved consent form, must be obtained before the participant is involved in any study-related procedure. The consent form must be signed and dated by the participant and/or the participant’s guardian or legally authorized representative, and by the person obtaining the consent. The participant must be given a copy of the signed and dated consent document. The original signed copy of the consent document must be retained in the medical record or research file.

## Study Documentation

The investigator must prepare and maintain adequate and accurate source documents designed to record all observations and other data pertinent to the study for each research participant. This information enables the study to be fully documented and the study data to be subsequently verified.

# DATA AND SAFETY MONITORING

The overall PI will be responsible for monitoring the trial per the trial monitoring plan, in addition to overseeing the safety and efficacy of the trial, executing the DSM plan and complying with all reporting requirements to the local and federal authorities. This oversight will be accomplished through additional oversight from the Data and Safety Monitoring Committee (DSMC) at the University of Colorado Cancer Center (CU Cancer Center).

## Data and Safety Monitoring Committee (DSMC)

The DSMC is responsible for ensuring data quality and patient safety for all clinical studies at the CU Cancer Center. A summary of the DSMC’s activities is as follows:

- Conduct internal audits
- Ongoing review of all serious adverse events (SAEs), unanticipated problems (UAPs) and reportable adverse events (AEs)
- Has the authority to close and/or suspend trials for safety or trial conduct issues
- May submit recommendations for corrective actions to the CU Cancer Center’s Executive Committee

All SAEs, UAPs and reportable AEs are to be reported to the DSMC within 5 business days of receiving notification of the occurrence. For all outside sites, these should be reported to the CHCO coordinator.

The overall PI will provide a DSM report to the CU Cancer Center DSMC on a six month basis. The DSM report will include a protocol summary; current numbers; summary of toxicity data to include specific SAEs, UAPs and AEs; any dose modifications; all protocol deviations; and protocol amendments. Results and recommendations from the review of this six month report by the DSMC will then be submitted by CHCO to COMIRB at continuing review.

**6 Month Safety Reports:** The PI will provide a DSM report to the CU Cancer Center DSMC on a six month basis. The DSM report will include a protocol summary; current enrollment numbers; summary of toxicity data to include specific SAEs, UAPs and AEs; any dose modifications; all protocol deviations; and protocol amendments. The DSM report to the DSMC will also include, if applicable, the results of any efficacy data analysis conducted, as well as any internal DSMB reports. Results and recommendations from the review of this six month report by the DSMC will then need to be submitted to the IRB of record at the time of continuing review.

## Data Collection

Minimal data will be collected for this study. Data outside of CHCO will be submitted to the CHCO coordinator for review with the overall PI.

| **Form / Source Documentation(SD)** | **Submission Timeline** |
| --- | --- |
| Eligibility Checklist Form with supporting SD | Prior to starting any procedures |
| Consent and Assent (SD) | Prior to starting any procedures |
| Sample Collection Form | To be sent with samples |
| Local Lab reports (SD) | Within 14 days of subject finishing treatment |
| Adverse Event Log | Within 7 days of subject finishing 30 day monitoring period |
| Clinic notes while on treatment (SD) | Within 14 days of subject finishing treatment |
| Follow-up Forms | Within 7 days of subject finishing 30 day monitoring period |

# DATA ANALYSIS PLAN

Patients whose tumors are determined to be consistent with DMG by pathological review will be considered evaluable.

For the primary aim, gemcitabine will be considered to be detectable in a patient tumor sample if gemcitabine, dFdU, or dFdCTP is detected at a measurable level in any of the samples run. Undetectable gemcitabine will be defined as gemcitabine, dFdU, and dFdCTP levels below the detectable limits for all samples run, in the setting of detectable gemcitabine, dFdU, or dFdCTP in the plasma and/or CSF for that patient, and the pathologic review showing viable tumor tissue.

For the secondary aim, gemcitabine, dFdU, and dFdCTP will be quantified in the available tumor sample(s), in plasma, and in CSF (if available). The tumor concentrations will be compared to historic controls in adult GBM. Tumor:plasma and tumor:CSF ratios will also be calculated.

# KNOWLEDGE TO BE GAINED

Through this study, we will measure drug penetration of systemic chemotherapy into human DMG tissue for the first time. By choosing a medication that we have shown to be effective against DMG *in vitro* and that penetrates the BBB, these findings will be as generalizable as possible to other systemic chemotherapies. Furthermore, we have several control drug levels, including a very similar study performed in adult GBM, against which to compare our penetration data. This study uses a small sample size, and minimal treatment apart from standard of care (one dose of gemcitabine), to provide novel evidence for or against continuing the use of systemic chemotherapy in DMG. Importantly, either outcome (detectable or undetectable gemcitabine) will be crucial and novel data to the field of DMG research.

# POTENTIAL SCIENTIFIC PROBLEMS

## Adequacy of Tissue Quantity for Analysis

We have worked with the Medical Chemistry Core Facility at UC-Denver, led by Dr. Michael Wempe, to determine the analytic methods planned and the minimal amount of tissue needed for determination of gemcitabine concentration. We have determined that the amount of tissue likely to be needed for quantification is approximately 10 mg, equivalent to approximately three of six available cores. If we are unable to quantify all of the metabolites from this quantity of tissue, or if the cores are unexpectedly small for a particular patient, several contingencies exist. There are two other mass spectrometers available in the core facility that have an approximately 10-fold greater sensitivity, so one of these machines could be used. These machines are not generally available for determining the quantification method and parameters, but these methods can be transferred easily from one machine to another to run samples if needed. Finally, dFdU concentrations tend to be higher than those for gemcitabine by approximately 100-fold, and concentrations of dFdU alone would be adequate to draw conclusions about gemcitabine penetration and compare to historic controls.

## Appropriateness of Controls

Since biopsy of normal pontine or other brain to serve as control tissue cannot be justified, we will use multiple other historic and patient-derived controls to allow analysis of our results. Because the primary aim of the study is to determine whether gemcitabine can penetrate DMG tissue, and the timing of treatment in this study is necessary to examine PK but excludes pharmacodynamic analysis, we will compare levels achieved to those in the study of gemcitabine in GBM (18), a tumor in which a measurable clinical effect from chemotherapy has been achieved; we will also compare levels to those found to be adequate to achieve a biologic effect in a preclinical model of gemcitabine treatment (25). We will also compare observed penetration to levels achieved in plasma and CSF (when available) to determine the tissue:plasma and tissue:CSF and plasma:CSF ratios, and to the IC_50_ levels we have determined for gemcitabine in a cell culture model of DMG in order to have a disease-specific measure of potential efficacy.

# REFERENCES

1. Fangusaro J. Pediatric high-grade gliomas and diffuse intrinsic pontine gliomas. Journal of child neurology. 2009;24(11):1409-17.

2. Perez-Gomez JL, Rodriguez-Alvarez CA, Marhx-Bracho A, Rueda-Franco F. Stereotactic biopsy for brainstem tumors in pediatric patients. Child's nervous system : ChNS : official journal of the International Society for Pediatric Neurosurgery. 2010;26(1):29-34.

3. Ogiwara H, Morota N. The efficacy of a biopsy of intrinsic brainstem lesions for decision making of the treatments. Child's nervous system : ChNS : official journal of the International Society for Pediatric Neurosurgery. 2013;29(5):833-7.

4. Puget S, Beccaria K, Blauwblomme T, Roujeau T, James S, Grill J, et al. Biopsy in a series of 130 pediatric diffuse intrinsic Pontine gliomas. Child's nervous system : ChNS : official journal of the International Society for Pediatric Neurosurgery. 2015;31(10):1773-80.

5. Wang ZJ, Rao L, Bhambhani K, Miller K, Poulik J, Altinok D, et al. Diffuse intrinsic pontine glioma biopsy: a single institution experience. Pediatric blood & cancer. 2015;62(1):163-5.

6. Kieran MW. Time to rethink the unthinkable: upfront biopsy of children with newly diagnosed diffuse intrinsic pontine glioma (DIPG). Pediatric blood & cancer. 2015;62(1):3-4.

7. Hankinson TC, Campagna EJ, Foreman NK, Handler MH. Interpretation of magnetic resonance images in diffuse intrinsic pontine glioma: a survey of pediatric neurosurgeons. Journal of neurosurgery Pediatrics. 2011;8(1):97-102.

8. Wu G, Broniscer A, McEachron TA, Lu C, Paugh BS, Becksfort J, et al. Somatic histone H3 alterations in pediatric diffuse intrinsic pontine gliomas and non-brainstem glioblastomas. Nature genetics. 2012;44(3):251-3.

9. Bechet D, Gielen GG, Korshunov A, Pfister SM, Rousso C, Faury D, et al. Specific detection of methionine 27 mutation in histone 3 variants (H3K27M) in fixed tissue from high-grade astrocytomas. Acta neuropathologica. 2014;128(5):733-41.

10. Reid JM, Qu W, Safgren SL, Ames MM, Krailo MD, Seibel NL, et al. Phase I trial and pharmacokinetics of gemcitabine in children with advanced solid tumors. Journal of clinical oncology : official journal of the American Society of Clinical Oncology. 2004;22(12):2445-51.

11. Steinherz PG, Seibel NL, Ames MM, Avramis VI, Krailo MD, Liu-Mares W, et al. Phase I study of gemcitabine (difluorodeoxycytidine) in children with relapsed or refractory leukemia (CCG-0955): a report from the Children's Cancer Group. Leukemia & lymphoma. 2002;43(10):1945-50.

12. Song BS, Seo J, Kim DH, Lim JS, Yoo JY, Lee JA. Gemcitabine and docetaxel for the treatment of children and adolescents with recurrent or refractory osteosarcoma: Korea Cancer Center Hospital experience. Pediatric blood & cancer. 2014;61(8):1376-81.

13. Rapkin L, Qayed M, Brill P, Martin M, Clark D, George BA, et al. Gemcitabine and docetaxel (GEMDOX) for the treatment of relapsed and refractory pediatric sarcomas. Pediatric blood & cancer. 2012;59(5):854-8.

14. Geoerger B, Chisholm J, Le Deley MC, Gentet JC, Zwaan CM, Dias N, et al. Phase II study of gemcitabine combined with oxaliplatin in relapsed or refractory paediatric solid malignancies: An innovative therapy for children with Cancer European Consortium Study. European journal of cancer. 2011;47(2):230-8.

15. Mora J, Cruz CO, Parareda A, de Torres C. Treatment of relapsed/refractory pediatric sarcomas with gemcitabine and docetaxel. Journal of pediatric hematology/oncology. 2009;31(10):723-9.

16. Cole PD, Schwartz CL, Drachtman RA, de Alarcon PA, Chen L, Trippett TM. Phase II study of weekly gemcitabine and vinorelbine for children with recurrent or refractory Hodgkin's disease: a children's oncology group report. Journal of clinical oncology : official journal of the American Society of Clinical Oncology. 2009;27(9):1456-61.

17. Wagner-Bohn A, Paulussen M, Vieira Pinheiro JP, Gerss J, Stoffregen C, Boos J. Phase II study of gemcitabine in children with solid tumors of mesenchymal and embryonic origin. Anti-cancer drugs. 2006;17(7):859-64.

18. Sigmond J, Honeywell RJ, Postma TJ, Dirven CM, de Lange SM, van der Born K, et al. Gemcitabine uptake in glioblastoma multiforme: potential as a radiosensitizer. Annals of oncology : official journal of the European Society for Medical Oncology / ESMO. 2009;20(1):182-7.

19. Bapiro TE, Richards FM, Goldgraben MA, Olive KP, Madhu B, Frese KK, et al. A novel method for quantification of gemcitabine and its metabolites 2',2'-difluorodeoxyuridine and gemcitabine triphosphate in tumour tissue by LC-MS/MS: comparison with (19)F NMR spectroscopy. Cancer chemotherapy and pharmacology. 2011;68(5):1243-53.

20. Schwartzentruber J, Korshunov A, Liu XY, Jones DT, Pfaff E, Jacob K, et al. Driver mutations in histone H3.3 and chromatin remodelling genes in paediatric glioblastoma. Nature. 2012;482(7384):226-31.

21. Barua NU, Lowis SP, Woolley M, O'Sullivan S, Harrison R, Gill SS. Robot-guided convection-enhanced delivery of carboplatin for advanced brainstem glioma. Acta neurochirurgica. 2013;155(8):1459-65.

22. Zhou Z, Ho SL, Singh R, Pisapia DJ, Souweidane MM. Toxicity evaluation of convection-enhanced delivery of small-molecule kinase inhibitors in naive mouse brainstem. Child's nervous system : ChNS : official journal of the International Society for Pediatric Neurosurgery. 2015;31(4):557-62.

23. Carceller F, Aleu A, Casasco A, Guimaraens L, Lopez-Pino MA, Madero L, et al. Superselective intracerebral catheterization for administration of oncolytic virotherapy in a case of diffuse intrinsic pontine glioma. Journal of pediatric hematology/oncology. 2014;36(7):e430-2.

24. DiResta GR, Lee JB, Arbit E. Measurement of brain tissue specific gravity using pycnometry. Journal of neuroscience methods. 1991;39(3):245-51.

25. Pauwels B, Korst AE, Lardon F, Vermorken JB. Combined modality therapy of gemcitabine and radiation. The oncologist. 2005;10(1):34-51.
