# Supplementary material for: Preclinical and clinical investigation of intratumoral chemotherapy pharmacokinetics in DIPG using gemcitabine
Source: Neurooncol Adv. 2020 Feb 24;2(1):vdaa021. doi: 10.1093/noajnl/vdaa021 (PMC7212907; doi:10.1093/noajnl/vdaa021)
Supplement: vdaa021_suppl_Supplementary_Material [file vdaa021_suppl_supplementary_material.docx]

**Full Methods**

*Cell culture*: Primary human pediatric DIPG/HGG cell lines (SU-DIPG-IV and SU-DIPG-VI, derived from previously irradiated DIPGs at autopsy, provided by Dr. Michelle Monje, Stanford University; SF7761, derived from a DIPG biopsy sample, provided by Dr. Nalin Gupta, University of California-San Francisco; BT-245, derived from a thalamic diffuse midline glioma at initial resection, provided by Dr. Keith Ligon, Dana-Farber Cancer Institute; HSJD-GBM-001, derived from a frontal glioblastoma at initial resection, and HSJD-DIPG-007, derived from a previously irradiated DIPG at autopsy, both provided by Dr. Angel Montero Carcaboso, Hospital Sant Joan de Deu) were grown in neurosphere (suspension) culture conditions in tissue culture-treated flasks (Falcon/Corning) (SF7761) or in ultra-low attachment flasks (Corning) (all others). In addition, U87 cells obtained from ATCC were grown as neurospheres in ultra-low attachment flasks. The identity of all lines was validated by microsatellite DNA profiling and compared to known results prior to and during this project. BT-245 was maintained in NeuroCult NS-A medium (Stemcell Technologies) supplemented with penicillin-streptomycin (1:100), heparin (2 µg/mL, Stemcell Technologies), human epidermal growth factor (EGF, 20 ng/mL, Shenandoah Biotech), human basic fibroblast growth factor (FGFb, 20 ng/mL, Shenandoah Biotech), and human platelet derived growth factors A and B (PDGFR-AB, 20 ng/mL, Shenandoah Biotech). SU-DIPG-IV, SU-DIPG-VI, and SF7761 were maintained in Neurobasal-A medium mixed 1:1 with Dulbecco's modified Eagle's medium/F-12 supplemented 1:100 by volume with HEPES [4-(2-hydroxyethyl)-1-piperazine ethanesulfonic acid] 1 M, sodium pyruvate 100 mM, MEM Non-Essential Amino Acids 10 mM, Glutamax-I, and antibiotic-antimycotic (all Gibco/Life Technologies); B27-A supplement 50x (1:50, Invitrogen); heparin (2 μg/mL, Stemcell Technologies); and human EGF, FGFb, and PDGFR-AB (all 20 ng/mL, Shenandoah Biotech). For SF7761 cells, N2 supplement was also added (1:100, Life Technologies). U87 cells were maintained in Neurobasal-A supplemented with EGF (20 ng/mL), FGF (20 ng/mL), B-27 (1:50) and heparin (2 μg/mL). Prior to all endpoint measurements or mouse injection, cells growing as neurospheres were dispersed by trituration using a micropipette or by treatment with TrypLE Express Enzyme (Gibco/Life Sciences).

*Gemcitabine dose-response curves:* Cells were plated in 96 well ultra-low attachment plates at a density of 20,000 cells per well in 100 µL medium. Cells (n=3 wells per condition) were then incubated in a range of concentrations of gemcitabine or in DMSO control for 72 hours (SU-DIPG-IV/SU-DIPG-VI) or 120 hours (SF7761/HSJD-DIPG-007), after which viability was determined by adding 20 µL/well of MTS reagent (CellTiter 96® AQueous One Solution Cell Proliferation Assay, Promega). Cells were incubated for 1-6h, during which period absorbance at 490 nM was measured using a plate reader (Synergy H1, BioTek).

*Orthotopic xenografts*: Athymic nude-Foxn1nu female mice (Charles River), aged 6 to 12 weeks, were used for intracranial xenografts. Mice were first randomized to cell line, location, and treatment timing groups. For tumor experiments, intracranial injection of U87, BT-245 and HSJD-GBM-001 cells suspended in 3 μL of PBS was stereotactically carried out under isoflurane anesthesia. For cortical injections, the coordinates were 2.5 mm right and 2.0 mm anterior to bregma, then 3.5 mm below the skull surface. For pontine injections, the coordinates were 1.0 mm right and 0.8 mm posterior to lambda, then 5.0 mm below the skull surface. Mice received 5 mg/kg of carprofen as analgesic for two consecutive days postoperatively. Animals were weighed twice weekly. Mice were treated at the time of initial tumor-related symptoms, generally ataxia, decreased movement, or 20% weight loss. All mice were treated with gemcitabine 120 mg/kg intraperitoneally (IP) and then sacrificed, by carbon dioxide inhalation and subsequent exsanguination via cardiac puncture, at each mouse’s predetermined time following treatment. Brains were then excised and divided into tumor (when applicable), normal pons, and normal cortex, then snap frozen in dry ice. Whole blood was collected by cardiac puncture. Plasma is then isolated via centrifugation and maintained at -80°C until processing. Numbers of mice per condition are included in Results and figure legends and reflect intention-to-treat analysis.

*Phase 0 clinical trial:* The study opened in September 2016; two patients have been screened and two enrolled to this point. Patients age 3-18 years with newly diagnosed DIPG, based on clinical symptoms and brain MRI findings consistent with the diagnosis in the opinion of the local multi-disciplinary neuro-oncology team, are eligible. The full study protocol is included in the supplemental materials. Other eligibility criteria include normal bone marrow, kidney, and liver function, as well as a Lansky/Karnofsky score ≥ 60. Exclusion criteria include prior tumor-directed treatment, and tumor or clinical features making surgical intervention unsafe in the opinion of the treating neurosurgeon. At our institution, stereotactic trans-cerebellar needle biopsy is offered at DIPG diagnosis as standard of care and is not considered part of the clinical trial protocol. Enrolled patients are administered gemcitabine 2,100 mg/m^2^ IV over 30 minutes, with no more than four hours allowed between the end of the infusion and obtaining of biopsy specimens to match the previous adult data and our mouse studies as closely as clinically feasible. Per institutional standard of care, eight needle core biopsies are taken from four separate quadrants of the tumor’s circumference at two depths. Four cores are reserved for pathological review and future biology studies, while four are available for study analysis. All cores are snap frozen and maintained at -80°C until processing. Peripheral blood is also drawn at the time of biopsy. Plasma is isolated via centrifugation and maintained at -80°C until processing. A summary of the intratumoral drug concentration findings is discussed with the family once available (within one month of surgery) to help with planning of subsequent therapy. Of note, the study has now been amended to include DMG outside the pons and recurrent DIPG/DMG requiring tumor resection or biopsy.

*Determination of gemcitabine concentration in tissue samples:* An Applied Biosystems Sciex 4000 (Applied Biosystems, Foster City, CA) was equipped with a Shimadzu HPLC (Shimadzu Scientific Instruments, Inc., Columbia, MD) and Leap auto-sampler (LEAP Technologies, Carrboro, NC). Gemcitabine hydrochloride was procured from Toronto Research Chemicals (Ontario, Canada), and stock DMSO concentrations were prepared. An internal control measurement for gemcitabine was determined using a variant gemcitabine compound enriched with Carbon 13. Gemcitabine concentrations were determined using a liquid chromatography mass spectrometry – mass spectrometry (LC/MS-MS) method employing a Thermo Scientific Hypersil Silica column (250 x 4.6 mm; 5 micron) run at 40^o^C with a flow rate of 0.4 mL/min. Solvent A was HPLC grade water with 10 mM ammonium acetate and 0.1% formic acid; solvent B was methanol:acetonitrile (1:1). Samples (10 µL) were injected. The gradient was as follows: 95% A held for 1.0 min and then ramped to 50% A at 4.0 min and held for 3.0 min, at which point was ramped to 95% B at 9.0 min and held for 5.0 min and ramped back to 95% A at 16.0 min and held for 9.0 min; total run of 25 min. Instrument settings included CAD = 12, GS1/GS2 = 30, Q1 and Q3 resolution set to Low, CXP = 6, IS = 5500, and Temp at 550^o^C. Gemcitabine had 264.1 🡪 112.1 m/z with DP = 76, CE = 25 and t_R_ = 11.0 min. Control human plasma was procured from BioIVT (Westbury, NY) and used to prepare a standard curve for gemcitabine (0.0005 – 1.0 µM) via serial dilution methods. Plasma (100 µL) samples were extracted with extraction solution (200 µL) containing IS. Gemcitabine R^2^ = 0.9972 with a LOQ = 0.52 ng/mL. Control mouse brain tissue was used to prepare standard curves. Tissue samples (mg) were homogenized using a handheld homogenizer with two volumes (w/v) of HPLC grade water and homogenate extracted with extraction solution containing IS; gemcitabine R^2^ = 0.9980.

*Statistical analysis:* Mean values for gemcitabine concentrations were compared via unpaired t-test. For dose-response curves, curve fitting and IC_50_ calculation were done via Graphpad Prism, and IC_90_ values were calculated at [www.graphpad.com/quickcalcs/ECanything2/](http://www.graphpad.com/quickcalcs/ECanything2/).

*Study approval:* The single-institution clinical trial was approved by the Colorado Multi-Institutional Review Board (COMIRB 15-1621, NCT02992015) at Children’s Hospital Colorado, and families of subjects completed informed consent before enrollment. Animal experiments were approved by the Institutional Animal Care and Use Committee at the University of Colorado Anschutz Medical Campus.
